# LP(A) AND METABOLIC SYNDROME IN OLDER PEOPLE

**DOI:** 10.1093/geroni/igac059.007

**Published:** 2022-12-20

**Authors:** Nikolaus Buchmann, Sabine Schipf, Till Ittermann, Elisabeth Steinhagen-Thiessen, Ilja Demuth, Marcello Markus

**Affiliations:** Charité - University Medicine Berlin, Berlin, Berlin, Germany; Institute for Community Medicine, University Medicine Greifswald, Germany, Greifswald, Mecklenburg-Vorpommern, Germany; Institute for Community Medicine, University Medicine Greifswald, Germany, Greifswald, Mecklenburg-Vorpommern, Germany; Charité - Universitätsmedizin Berlin, Berlin, Berlin, Germany; Charité Universitätsmedizin Berlin, Berlin, Berlin, Germany; Department of Internal Medicine B, University Medicine Greifswald, Greifswald, Germany, Berlin, Mecklenburg-Vorpommern, Germany

## Abstract

**Background:**

Although an inverse association between type II diabetes mellitus and Lipoprotein (a) [LP(a)] has already been well researched, there is sparse data on the association between metabolic syndrome (MetS) with Lp(a). MetS is highly prevalent in older people and insulin resistance in MetS might link Lp(a) with MetS. Thus, we analyzed the association between Lp(a) with MetS in two large cohorts the Berlin Aging Study II [BASE-II] and the Study of Health in Pomerania [SHIP-0]

**Methods:**

Complete cross-sectional data was available for 5,743 BASE-II and SHIP-0 participants (48.7% men; age 58 [20-85] years). MetS was defined according to modified criteria of the AHA/NHLBI 2009 definition. The association between MetS with Lp(a) was examined by median regression adjusted for age, sex and study and models were stratified by gender and menopause.

**Results:**

MetS was prevalent in 27.6% (n=1,573) participants. We found an inverse association between MetS with Lp(a) in the whole study sample (β=-11.9 [95% confidence interval (95% CI) -21.3 to -2.6]) as well as in men (β=-16.5 [95% CI -28.6 to -4.3]) and in postmenopausal women (β=-25.4 [95% CI -46.0 to -4.8]). In contrast to this, in premenopausal women a positive association between MetS and Lp(a) (β=39.2 [95% CI 12.3 to 65.9]) was evident.

**Conclusion:**

Postmenopausal changes in hormone metabolism impact both MetS and Lp(a). With respect to the ongoing development of Lp(a)-lowering drugs, their use must be examined, particularly in old age and in subjects with MetS.

